# 

**Published:** 1906-04

**Authors:** 


					﻿____	!	W-ftm0!1 ?! o<?.
A___A TP	PUBLISHED MONTHLY.
JOURNAL-WORD
/r,k;;rOF MEDICO-
Successor to Atlanta riedlcal and Surgic|^Jo#£?ratf B^tabfiiheA-iPB^? I
and Southern riedlcal Record/.Established 1870.
*	F MIG M.19Q7	1
Vol. VIII.	Atlanta, Ga., Apr^l, 1906	‘I
				

